# Supplementary material for: Unidirectional hybridization between American paddlefish Polyodon spathula (Walbaum, 1792) and sterlet Acipenser ruthenus (Linnaeus, 1758)
Source: PeerJ. 2024 Jan 19;12:e16717. doi: 10.7717/peerj.16717 (PMC10802154; doi:10.7717/peerj.16717)
Supplement: Supplemental Information 1 — Percentage of fertilization = number of live eggs at neurulation/100 eggs; percentage of survival = number of live fish at 30 days post hatch/100 eggs. [file peerj-12-16717-s001.docx]

**Supplemental 1 Experimental design and embryo development with percentage of fertilization and survival of hybrids (sterlet *Acipenser ruthenus* x American paddlefish *Polyodon spathula*) and purebred families.**

|  | Parent  combinations | Fertilization % | Survival  % | Embryo development |
| --- | --- | --- | --- | --- |
| Female sterlet x Male paddlefish | ST1 x PF1 (n=1010) | 44±5 | 26±2 | viable hybrid fish |
|  | ST1 x PF2 (n=944) | 56±7 | 21±2 |  |
|  | ST2 x PF1 (n=670) | 19±8 | 15±4 |  |
|  | ST2 x PF2 (n=604) | 20±7 | 18±2 |  |
| Female paddlefish x Male sterlet | PF3 x ST3 | Not determinable | 0 | arrest of development at gastrula/early neurula stage |
| Sterlet x Sterlet | ST1 x ST4 | 85±3 | 70±8 | viable sterlet |
|  | ST2 x ST4 | 68±6 | 56±6 |  |
| Paddlefish x Paddlefish | PF4 x PF1 | 55±5 | 53±8 | viable paddlefish |
|  | PF4 x PF2 | 62±9 | 46±4 |  |
|  | PF3 x PF1 | 70±11 | 61±7 |  |

Percentage of fertilization = number of live eggs at neurulation/100 eggs;

percentage of survival = number of live fish at 30 days post hatch/100 eggs.
